# Occurrence, Diversity of *Listeria* spp. Isolates from Food and Food-Contact Surfaces and the Presence of Virulence Genes

**DOI:** 10.3390/microorganisms8020294

**Published:** 2020-02-20

**Authors:** Franca Rossi, Carmela Amadoro, Daniele Conficoni, Valerio Giaccone, Giampaolo Colavita

**Affiliations:** 1 Istituto Zooprofilattico Sperimentale dell’Abruzzo e Molise (IZSAM), Teramo, Diagnostic Laboratory, 86100 Campobasso, Italy; f.rossi@izs.it; 2 Dipartimento di Medicina e Scienze della Salute “V. Tiberio”, Università degli Studi del Molise, 86100 Campobasso, Italy; colavita@unimol.it; 3 Dipartimento di Medicina Animale, Produzioni e Salute, Università degli Studi di Padova, Viale dell’Università, 16, 35020 Legnaro, Italy; daniele.conficoni@gmail.it (D.C.); valerio.giaccone@unipd.it (V.G.)

**Keywords:** *Listeria* spp., food, food-contact surfaces, genotyping, virulence genes, toxin–antitoxin system *maz*EF

## Abstract

This study evaluates the hazards posed by foodborne bacteria of the *Listeria* genus by analyzing the occurrence, diversity and virulence of *Listeria* spp.in food and food-manufacturing plants. Seventy-five isolates obtained from the routine analysis of 653 samples taken by three diagnostic laboratories in Northern Italy were genotypically differentiated by Repetitive Extragenic Palindrome (rep) PCR, with the GTG5 primer identified by sequencing the 16S rRNA gene and examined by specific PCR tests for the presence of *L. monocytogenes* virulence determinants occasionally found to occur in other species of the genus. Within this sample, 76% (*n* = 57) isolates were identified as *L. innocua*, 16% (*n* = 12) as *L. monocytogenes*, 6.6% (*n* = 5) as *L. welshimeri* and 1.3% (*n* = 1) as *L. seeligeri*. All *L. monocytogenes* isolates belonged to the serotype 1/2a and were predicted to be virulent for the presence of the *inl*J internalin gene. Potentially virulent strains of *L. innocua*, *L. seeligeri* and *L. welshimeri*, carrying the *L. monocytogenesinl*A gene and/or *hly* gene, were identified, and most isolates were found to possess the toxin–antitoxin system *maz*EF for efficient adaptation to heat shock. Results indicated the need to reinforce food-contamination-prevention measures against all *Listeria* species by defining efficiently their environmental distribution.

## 1. Introduction

Bacteria belonging to the low-G+C facultatively anaerobic, Gram-positive *Listeria* genus are ubiquitous and can contaminate food products of both animal and plant origin [1].

The human pathogen *Listeria monocytogenes* can cause severe foodborne infections, including septicemia, meningitis and endocarditis, with fatality rates of 20–30% in the elderly, immunocompromised persons and pregnant women, with infection in the latter resulting in abortion or neonatal infections [2]. It grows at refrigeration temperatures, under vacuum or in a modified atmosphere, tolerates low-pH values and forms sanitation-resistant biofilms in food-processing plants. These characteristics make contamination prevention the main defense against this pathogen [3]. Ready-to-eat (RTE) foods are those with higher risk to transmit the bacterium. In these products, *L. monocytogenes* must not reach levels higher than 100 CFU/g during their shelf life, while it must be absent from foods intended for infants or special medical purposes and from foods with a_w_ and pH values that allow the growth of the bacterium up to the limit of 100 CFU/g during their shelf life [4].

Beyond *L. monocytogenes*, *L. ivanovii* is pathogenic for animals and humans [5]. In addition, illnesses caused by virulent strains of *L. innocua* and *L. seeligeri* [6,7,8] have been reported. Meningitis was caused by an *L. innocua* strain possessing *L. monocytogenes* internalin genes *inl*A and *inl*B and genes *prf*A, *hly* and *plc*A of the pathogenicity island 1 (LIPI1) [8], considered relics left over after divergence from *L. monocytogenes* [9]. Atypical *L. innocua* strains possess different combinations of *L. monocytogenes* LIPI-1, *inl*A and *inl*B genes [10]. Strains harboring only LIPI-1 are hemolytic but not virulent, while those expressing *inl*A enter non-phagocytic-epithelial cells expressing *inl*A receptor E-cadherin (Ecad) as efficiently as *L. monocytogenes*. However, *L. innocua* expressing *inl*A, but not LIPI-1 genes, was not able to escape from the vacuole and polymerize actin in the cytosol, and was, therefore, less virulent than their counterpart expressing LIPI-1 genes in a mouse model [10]. Other hemolytic *L. innocua* strains possess the *L. monocytogenes* pathogenicity island LIPI-3, comprising the hemolysin S*lls*A gene, frequently found in lineage I of *L. monocytogenes* [11].

Not much is known about the occurrence of virulent *Listeria* spp. other than the *L. monocytogenes* strains in food; hence, this study was carried out to analyze the occurrence, diversity and virulence traits of *Listeria* spp. isolated during routine analysis of food products and swabs from production-plant surfaces in contact with food.

Molecular typing by rep-PCR with the GTG5 primer, allowing rapid *L. monocytogenes*-strain differentiation [12], was applied to selected representative isolates to be identified by 16S rRNA gene sequencing.

The presence of the *L. monocytogenes* genes *inl*A and of *hly* and *lss*A—indicators of *L. monocytogenes* LIPI-1 and LIPI-3 presence, respectively—was analyzed in all *Listeria* spp. strains except from the *L. monocytogenes* isolates, to identify potentially virulent strains.

The *L. monocytogenes* isolates were identified at the serotype level by the multiplex PCR test described by Chen and Knabel in 2007 [13], and their virulence was assessed based on the presence of the putative internalin gene *inl*J, corresponding to locus *lmo2821* in the complete genome of *Listeria monocytogenes* EGD-e (acc.no. AL591824), which is invariably associated with virulence in mice [14].

An analysis of the distribution of the *maz*F gene, a component of the type II toxin–antitoxin system *maz*EF, conferring increased tolerance to heat shock [15], was carried out to obtain indications of the tolerance of the isolates to a key stress factor applied in food production.

## 2. Materials and Methods

### 2.1. Bacterial Strains and Culture Conditions

Reference strains *L. monocytogenes* ATCC 7644 and *L. innocua* ATCC 33090 were used for genotypic profile comparison. In this study, 102 presumptive *Listeria* spp. isolates, obtained in the years 2016–2017 from three diagnostic laboratories (Lab 1, 2 and 3) that analyzed 653 samples of food and food-plant surfaces in contact with food in Northern Italy, according to the UNI EN ISO norm 11290 Parts 1 and 2, Version 2005, were characterized. The isolates were previously identified physiologically with the standardized system API® *Listeria* (Biomerieux Italia, Bagno a Ripoli, FI, Italy). The sources of the strains are reported in Table 1 and each isolate originated from a different sample. Intentionally, a sampling strategy was avoided to delineate a realistic exposure scenario. The samples analyzed by Laboratories 1, 2 and 3 were each conferred by a different food-business operator. Their number per category is reported in Table 1. The 102 presumptive *Listeria* spp. isolates examined initially were numbered from 1 to 30, from 31 to 69, and from 70 to 102 for Laboratories 1, 2 and 3, respectively, but only those confirmed to belong to *Listeria* spp. by genetic tests are reported in Table 1. 

All bacterial strains were streaked onto blood agar (Liofilchem, Roseto degli Abruzzi, TE, Italy) plates to obtain isolated colonies. In this way, isolates were also tested for hemolytic activity. Single colonies were sub-cultured in tubes of Tryptic Soy (TS) broth (Biolife Italiana, Milan, Italy). Cultures were incubated in aerobiosis at 37 °C for 18–24 h. For long-term storage, the cultures were kept at −20 °C in the same medium with the addition of 20% glycerol.

### 2.2. DNA Extraction

DNA was extracted from 2 mL of fresh culture using the Genomic DNA Extraction Kit RBC Bioscience (Diatech Labline, Jesi, AN, Italy), according to the manufacturer instructions. The quantity and integrity of the extracted DNA were checked by comparison with known amounts of Lambda DNA (ThermoFisher Scientific, Rodano, MI, Italy) and for the absence of DNA smears on 1.5% *w*/*v* agarose gel in 1× TAE buffer (80 mM Tris-acetate, 2 mM EDTA, pH 8.0) at 120 V stained with 1:10,000 diluted GelRed (Biotium, Società Italiana Chimici, Rome, Italy).

### 2.3. PCR Tests

PCR primers used in this study, respective targets and references are reported in Table 2. The amplification conditions were those described in the cited references.

Primers *maz*F_qPCR_Fw and *maz*F_qPCR_rv were evaluated by BLASTn (https://blast.ncbi.nlm.nih.gov) and Clustal Omega (https://www.ebi.ac.uk/Tools/msa/clustalo/) for their ability to target *maz*F homologs in *Listeria* species other than *L. monocytogenes*. The amplification conditions were those reported by Curtis et al. [15].

All of the PCR tests were carried out with the EmeraldAmp GT PCR Master Mix Takara Clontech (Diatech, Jesi, Italy) under the conditions described in the respective literature (Table 2). The amplification products were separated on 1.5% (*w*/*v*) agarose gels.

### 2.4. Numerical Analysis of Genotypic Profiles

Genotypic profiles obtained by rep-PCR were analyzed by the BioNumerics V5.10 software (Applied-Maths, Belgium), using the Dice coefficient for pairwise comparison, and the Unweighted Pair Group Method, using Arithmetic Averages (UPGMA) clustering.

### 2.5. Sequencing and Sequence Analyses

Before sequencing, the PCR products were purified using the HiYield Gel/PCR Fragment Extraction Kit RBC Bioscience (Diatech) according to the instructions. The sequencing of both amplicon strands was carried out by Eurofins Genomics (Ebersberg, Germany), with the same primers used for amplification. Sequencing was carried out for species-level identification based on 16S rRNA gene identity and to confirm the PCR product identity in targeted assays of *inl*A, *inl*J, *hly*, *lls*A and *maz*F genes by BLASTn. Sequencing of the 16S rRNA gene was carried out for one isolate from each rep-PCR cluster separated at 85% similarity, a cut-off value chosen on the basis of the lowest similarity exhibited by ten duplicate strains in preliminary experiments.

## 3. Results

### 3.1. Typing and Identification of Listeria spp. Isolates

**Figure 1:**
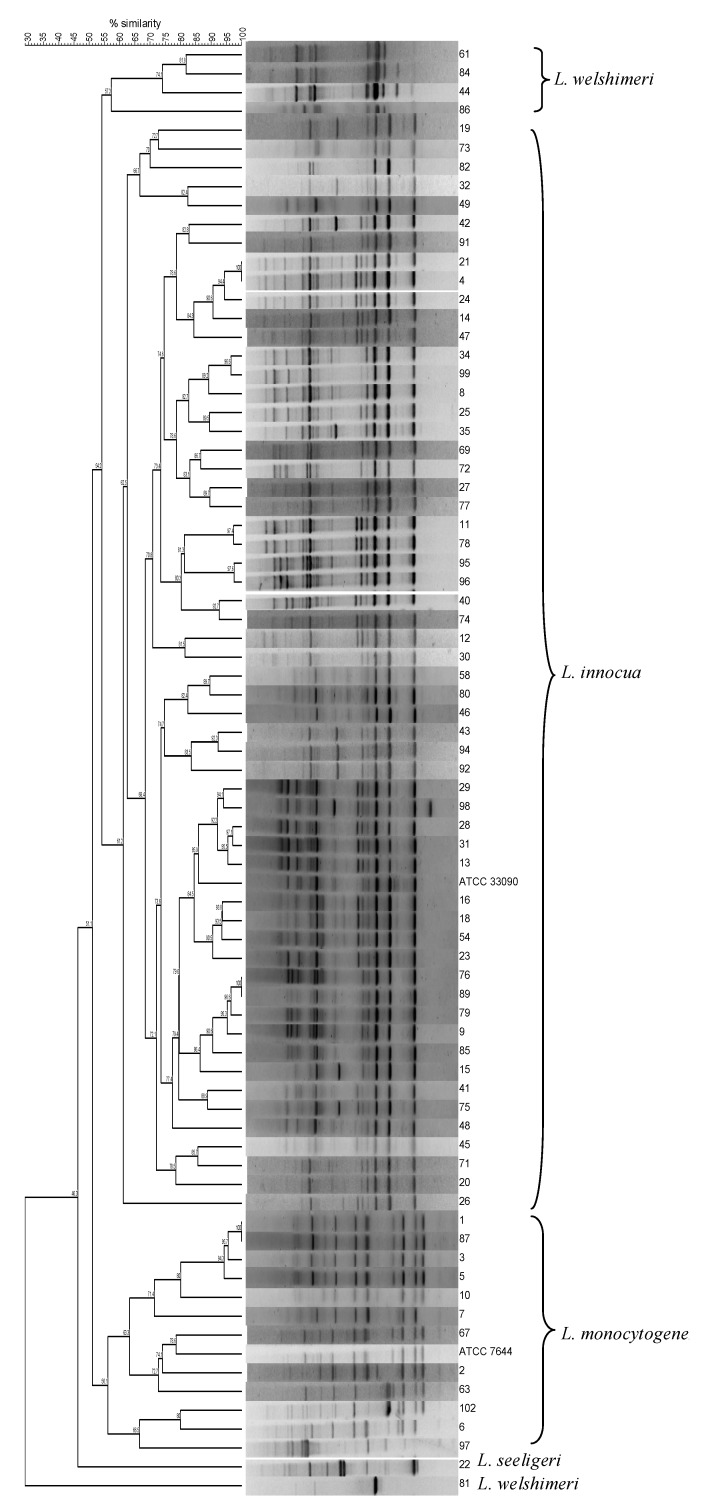
Clustering of *Listeria* spp. isolates from food and food-production plants based on rep-PCR profiles.

Numerical analysis of rep-PCR profiles enabled the distinction of three main clusters exhibiting comparable internal diversity, each comprising exclusively of *L. innocua*, *L. monocytogenes* or *L. welshimeri* isolates, as ascertained by sequencing the 16S rRNA gene, with the exception of *L. welshimeri* 81 that fell outside the group formed by the other *L. welshimeri* isolates. It was observed that the rep-PCR profile of this strain lacked most of the weaker bands observed in the other *L. welshimeri* profiles, exhibiting only a strong band common to those profiles. One isolate that did not belong to any of the three main clusters was identified as *L. seeligeri* (Figure 1). In all cases, identification at the species-level was based on 99% sequence identity with entries in the public domain database.

From Figure 1 and Table 1, it can be observed that isolates deriving from the same sample type showed less than 85% profile similarity, indicating that the *Listeria* spp. subtypes in the same food category differed. Conversely, highly similar genotypes were shown by isolates from different food categories.

Isolate 5 from a sample of raw sausage appeared highly similar to Isolates 1 and 3 from fresh pork (Figure 1, Table 1).

Based on genotypic identification, *Listeria* species were present in 34 out of 257 bovine meat samples, 5 out of 61 pork meat samples, 3 out of 45 fresh fish samples, 6 out of 43 raw cured pork product samples, 6 out of 36 frozen pizza samples, 3 out of 29 pizza dough samples, 3 out of 23 frozen pasta samples, 2 out of 15 roasted peppers samples, 9 out of 97 cheese-making plant swabs, and 6 out of 48 cheese-aging board swabs.

*L. monocytogenes* was detected in four fresh bovine meat samples, four fresh pork meat samples, two raw sausages, and two swabs from cheese-aging boards.

Variability in *Listeria* spp. occurrence among the three laboratories for different sample categories indicated an uneven distribution of these bacteria among sampling locations (Table 1).

### 3.2. Pathogenic Potential of the Listeria spp. Isolates

All of the *L. monocytogenes* isolates were attributed to serotype 1/2a, although none belonged to the major epidemic clone ECIII included in this serotype. *L. monocytogenes* isolates exhibited β-hemolysis and were all positive for the *inl*J-gene-targeted PCR assay, applied only to the *L. monocytogenes* isolates, being, therefore, classifiable as virulent according to Liu et al. 2003 [14].

*Listeria* isolates not belonging to the *L. monocytogenes* species were tested for the presence of *L. monocytogenes inl*A, *hly* and *lls*A genes. These were found in seven isolates (9.3% of all isolates). An amplification product with 100% sequence identity with *L. monocytogenes inl*A was obtained from *L. innocua* 74, *L. seeligeri* 22, and *L. welshimeri* 86. The *L. monocytogenes hly* gene was detected in *L. innocua* 23, 45, and 95 and *L. welshimeri* 86 and 98. Notably, *L. welshimeri* 86, endowed with both *L. monocytogenes inl*A and *hly* genes, could be highly virulent according to Moura et al. 2019 [10]. However, as all the *Listeria* spp. isolates, except for *L. monocytogenes* isolates, it was not hemolytic, indicating either the inability to express *hly* or that the gene product is not functional. The *lls*A gene was not detected in any *Listeria* spp. other than the *L. monocytogenes* isolate.

The PCR test for the presence of the *maz*EF toxin–antitoxin system gave an amplification product for all three species tested, though one or two discrepancies were present in the forward-primer-annealing site and three discrepancies were present in the reverse-primer-annealing site for all the species other than the *L. monocytogenes*. The *maz*F gene appeared to be absent in *L. innocua* isolates 31, 35 and 80 and to be most likely mutated at the primer-annealing sites for *L. monocytogenes* isolates 6 and 10, for which amplification was inefficient with the *maz*F-specific PCR test. However, most isolates were shown to possess this additional stress-adaptation system.

## 4. Discussion

This study presents the results of *Listeria* spp. occurrence based on the analysis of hundreds of food products and food-contact surfaces in manufacturing plants, and, as such, provides an insight into the distribution of these bacteria and to consumer exposure. With differences among the food categories, *Listeria* spp. appeared to be frequent in all food matrices and production plants, indicating that safety measures to prevent contamination must be improved, especially since innocuous species are considered indicators of *L. monocytogenes* co-occurrence [18].

A discrepancy between physiologic and molecular identification was observed in this study, since 27 of the 102 presumptive *Listeria* spp. provided by the laboratories were not confirmed to belong to the *Listeria* genus. It must be underlined that this constitutes a problem for the detection and enumeration of *Listeria* spp. in foods, since the EN ISO Standards 11290-1 and -2 often make it difficult to efficiently detect and enumerate *Listeria* spp. apart from *L. monocytogenes* in foods [19]. In particular, in this study, some presumptive *Listeria* spp. were genotypically identified as *Bacillus* spp. and *Enterococcus* species.

The application of rep-PCR with the GTG5 primer to different species in this study highlighted the usefulness of this technique in differentiating isolates of *L. monocytogenes*, *L. innocua*, and *L. welshimeri*. Its application in new studies regarding different *Listeria* species could lead to the definition of profile types to be used as references for a rapid preliminary identification of new isolates at the species-level, simultaneously allowing intra-species clustering. The possible correspondence of the intra-species clusters defined by rep-PCR with intra-species genetic variants should be investigated. The intra-species relationships defined by this genotyping method showed that highly similar genotypes belong to isolates from different food categories, meaning that some *Listeria* spp. subtypes are not associated with specific food products and are distributed across diverse environments, purportedly for their better ability to persist in processing plants. These subtypes should be thoroughly characterized genotypically and physiologically and preferentially used to test the efficiency of sanitation procedures.

An *L. monocytogenes* subtype, possibly derived from fresh meat, was identified in raw fermented sausages, an RTE food category. The highly similar isolates found in pork meat and sausages might represent a *L. monocytogenes* subtype able to survive in ready-to-eat cured pork products and should be investigated with genotyping methods with a higher resolution. A genetically closely related strain was isolated from bovine meat, indicating that the subtype comprising these isolates is distributed in different environments and is tolerant to the sausage-ripening process. This highlighted that the risk of *L. monocytogenes* growth in raw cured pork products should be assessed and that more efforts must be devoted to preventing the presence of *L. monocytogenes* in pork meat and processing plants by identifying specific contamination routes.

The monitoring of raw meat for the presence of *L. monocytogenes* through the application of genotyping methods with high discrimination power could elucidate whether this is the origin of processing-plant contamination, or whether inappropriate production and cleaning procedures favor the persistence of environmental *L. monocytogenes* subtypes in manufacturing plants. A similar approach should be applied to all RTE food categories in order to reduce contamination risk.

The isolation of *L. monocytogenes* serotype 1/2a in this study is in agreement with reports of its wide distribution in food and food-processing environments [12,20,21] and the consequently numerous listeriosis outbreaks that have, until recently, been caused by this serotype [22,23,24,25,26].

In this investigation, the genetic analysis of virulence characters highlighted that food can be a source of potentially pathogenic strains of *Listeria* spp. belonging to species generally considered to be innocuous. It was found that the *L. monocytogenes inl*A and *hly* virulence determinants can be harbored not only, as previously reported, by atypical *L. innocua* strains, but by *L. welshimeri* and *L. seeligeri* isolates as well. Therefore, species identification is not sufficient to estimate the risk associated with the presence of *Listeria* spp. in food, and both contamination prevention and the identification of contamination sources should be extended to all *Listeria* species. Further studies are needed to elucidate if the virulence genes found in this study in *Listeria* strains belonging to species considered commonly to be innocuous are functional.

The presence in most isolates of the *maz*EF toxin–antitoxin system, which indicates efficient heat-shock-adaptation capacity [15], underlines the need to reinforce contamination prevention to reduce the risk that these bacteria pose to consumers.

## Figures and Tables

**Table 1:** Number of samples analyzed by each laboratory, sample categories and respective *Listeria* spp. isolates as identified by molecular assays in this study. The *L. monocytogenes* virulence genes found in isolates of other *Listeria* species are reported adjacent to the respective isolates.

Sample Category	Lab1		Lab2		Lab3	
	n. Samples	Positive Samples/Isolates	n. Samples	Positive Samples/Isolates	n. Samples	Positive Samples/Isolates
Bovine meat	102	*L. innocua* 8, 9, 11, 12, 13, 16, 18, 21, 23 (*hly*), 24, 26, 27; *L. monocytogenes* 10	57	*L. innocua* 42, 43, 46	98	*L. innocua* 79, 80, 82, 85, 89, 91, 92, 94, 95 (*hly*), 96, 98 (*hly*), 99; *L. monocytogenes* 87, 97, 102; *L. welshimeri* 81, 84, 86 (*inl*A, *hly*)
Pork meat	31	*L. innocua* 4; *L. monocytogenes* 1, 2, 3, 7	9	0	21	0
Fresh fish	26	*L. innocua* 14, 15, 20	5	0	14	0
Raw cured pork products	16	*L. monocytogenes* 5, 6	13	*L. welshimeri* 44	14	*L. innocua* 73, 75
Frozen pizza	14	*L. innocua* 19, 25	13	0	8	*L. innocua* 71, 72, 76, 77
Pizza dough	5	0	2	*L. innocua* 32, 49	22	*L. innocua* 78
Frozen pasta	4	0	11	*L. innocua* 40, 41	8	*L. innocua* 74 (*inl*A)
Roast peppers	3		12	*L. innocua* 34, 35	0	0
Swabs from cheese-making plants	53	*L. innocua* 28, 29, 30; *L. seeligeri* 22 (*inl*A)	19	*L. innocua* 31, 45 (*hly*), 47, 48	25	0
Cheese-aging boards	22	0	12	*L. innocua* 54, 58, 69; *L. monocytogenes* 63, 67; *L. welshimeri* 61	14	0

The numbers missing from the series correspond to isolates for which identification as *Listeria* spp. was not confirmed by genotypic tests. The number of *Listeria* spp. isolates correspond to the number of positive samples.

**Table 2:** PCR primers used in this study, respective targets, amplicon size and references.

Primers	Sequence (5’→3’)	Usage	Amplicon Size (bp)	Reference
27f 1492r	AGAGTTTGATCMTGGCTCAG TACGGYTACCTTGTTACGACTT	16S rRNA gene amplification	1494	[16]
GTG_5_	GTGGTGGTGGTGGTG	Rep-PCR genotyping	n.a.	[12]
4bF 4bR	AGTGGACAATTGATTGGTGAA CATCCATCCCTTACTTTGGAC	identification of the *L. monocytogenes* serotype 4b	597	[13]
12aF 12aR	GAGTAATTATGGCGCAACATC CCAATCGCGTGAATATCGG	identification of the *L. monocytogenes* serotype 1/2a	724	[13]
ECIF ECIR	AATAGAAATAAGCGGAAGTGT TTATTTCCTGTCGGCTTAG	identification of the *L. monocytogenes* epidemic clone ECI	303	[13]
ECIIF ECIIR	ATTATGCCAAGTGGTTACGGA ATCTGTTTGCGAGACCGTGTC	identification of the *L. monocytogenes* epidemic clone ECII	889	[13]
ECIIIF ECIIIR	TTGCTAATTCTGATGCGTTGG GCGCTAGGGAATAGTAAAGG	identification of the *L. monocytogenes* epidemic clone ECIII	497	[13]
*hly*F *hly*R	CATTAGTGGAAAGATGGAATG GTATCCTCCAGAGTGATCGA	detection of the *L. monocytogenes hly* gene	730	[17]
*inl*AF1 *inl*AR1	TAACATCAGTCCCCTAGCAGGT TAGCCAACCTGTCACTATTGGA	detection of the *L. monocytogenes inl*A gene	516	[9]
*lmo*2821F *lmo*2821R	TGTAACCCCGCTTACACAGTT TTACGGCTGGATTGTCTGTG	detection of the *L. monocytogenes inl*J gene	611	[14]
llsAFor llsARev	CGATTTCACAATGTGATAGGATG GCACATGCACCTCATAAC	detection of *L. monocytogenes lls*A gene	280	[11]
*maz*F_qPCR_Fw *maz*F_qPCR_Rv	ACGGCCTGTTCTCATCATTC CGTTGGCAATTTTGCTTTTT	detection of the Listeria spp. *maz*F gene	103	[15]
